# Development of a Screening Tool for Feet/Footwear- Related Influences on Fall Risk

**DOI:** 10.1093/geroni/igab046.2898

**Published:** 2021-12-17

**Authors:** Mariana Wingood, Jennifer Vincenzo, Elizabeth Peterson, Christopher Neville

**Affiliations:** 1 University of Vermont, University of Vermont, Vermont, United States; 2 University of Arkansas for Medical Science, Fayetteville, Arkansas, United States; 3 University of Illinois Chicago, Champaign, Illinois, United States; 4 Upstate Medical University, Syracyse, New York, United States

## Abstract

The effectiveness of multifactorial fall risk assessment and intervention strategies is well documented. Although identifying feet/footwear-related influences on fall risk is a vital fall risk assessment component, few evidence-based resources or screening tools are available. To address this need, we developed the Screening Tool for Feet/Footwear-Related Influences on Fall Risk. Our tool is designed for older adults who are identified as at risk for falling, based on the CDC’s Stopping Elderly Accidents, Deaths, and Injuries (STEADI) Algorithm for Fall Risk Screening, Assessment, and Intervention. Tool development was informed by results of our systematic review of lower-limb factors associated with balance and falls. Our initial tool was evaluated by an external group of 9 interprofessional content experts. Those experts recommended modification of 8 items and rated the tool’s clarity as 81.2/100, appeal as 79.1/100, and clinical feasibility as 76.1/100. After incorporating recommended changes, we completed a modified Delphi study using 8 new interprofessional experts (average years of experience: 19.3). During Phase 1, Delphi participants recommended we combine items with similar treatment recommendations, add a question about orthoses, and increase the specificity of 9 items. This refinement resulted in a 20-item screening tool, which met approval after two rounds of consensus voting. Approval was defined based on the Item Content Validation Index, percentage of agreement > 80% on each item. The high level of agreement illustrates the tool’s content validity. Using our tool, an older adult’s feet/footwear-related risk factors can be identified and incorporated into an effective multifactorial fall prevention intervention.

